# Protocol-based management of older adults with hip fractures in Delhi, India: a feasibility study

**DOI:** 10.1186/s40814-016-0056-0

**Published:** 2016-03-09

**Authors:** Lalit Yadav, Abha Tewari, Anil Jain, Beverley Essue, David Peiris, Mark Woodward, Prakash Kotwal, Richard Lindley, Stephen Jan, Tracey Chantler, Premila Webster, Robyn Norton, Santosh Rath

**Affiliations:** 1 The George Institute for Global Health, 219-221, Splendor Forum, Plot No. 3, Jasola District Centre, New Delhi, 110025 India; 2 Department of Orthopaedics, University College of Medical Sciences, New Delhi, India; 3 The George Institute for Global Health, Sydney, Australia; 4 Primary Health Care Research, The George Institute for Global Health & Sydney Medical School, University of Sydney, Sydney, Australia; 5 Epidemiology & Biostatistics, The George Institute for Global Health & Statistics & Epidemiology, University of Oxford, Oxford, UK; 6 Department of Orthopaedics, All India Institute of Medical Sciences (AIIMS), New Delhi, India; 7 The George Institute for Global Health & Geriatric Medicine, Sydney Medical School, University of Sydney, Sydney, Australia; 8 Health Economics, The George Institute for Global Health & Sydney Medical School, University of Sydney, Sydney, Australia; 9 London School of Hygiene & Tropical Medicine, London, UK; 10 Nuffield Department of Population Sciences, School of Public Health, University of Oxford, Oxford, UK; 11 The George Institute for Global Health, University of Oxford, Oxford, UK; 12 The George Institute for Global Health, University of Sydney, Sydney, Australia; 13 Global Surgery, The George Institute for Global Health, University of Oxford, Oxford, OX1 3BD UK

**Keywords:** Feasibility, Hip fracture, Older adults, Protocol-based care, Mixed methods

## Abstract

**Background:**

Worldwide hip fractures are projected to increase from 1.7 million in 1990 to 6.3 million in 2050. In India, conservative estimates suggest an annual incidence of 600,000 osteoporotic hip fractures and this is expected to increase significantly due to ageing and increase life expectancy. Protocol-based ‘care pathways’ for the management of adults, over 60 years of age, with hip fractures in high-income countries has resulted in decreased mortality rates, early hospital discharge, improved quality of life and reduction in healthcare costs. The study objectives are to determine appropriateness, acceptability and feasibility of adopting best-practice guideline or protocol-based care for the management of hip fractures among older adults in India. The study will also identify barriers and facilitators in recruiting patients and retention till the agreed follow-up period.

**Methods:**

This will be a mixed-methods prospective cohort study. The quantitative data collection will involve recruitment of consecutive patients aged >50 years with an X-ray-confirmed hip fracture admitted in four tertiary care hospitals in Delhi, India, over a 2-month period. The quantitative data will be collected at three points: from patients at admission to hospital, from medical records at discharge and by telephone interviews with patients at 30 days post hip fracture. Qualitative data collection will involve key informant interviews, conducted with clinical leads and focus group discussions, conducted with groups of healthcare providers and patients and/or their carers. COM-B theoretical framework (capability, opportunity, motivation and behaviour) will be used to explore healthcare providers’ behaviour in order to facilitate development and implementation of appropriate integrated care pathway for management of older adults with hip fractures in India.

**Discussion:**

The proposed study will identify gaps in best practice in the management of older people with hip fractures in tertiary care hospitals in Delhi and document barriers and facilitators to the implementation of protocol-based care through recording the contextual realities of the health systems and care-seeking behaviours. Insights into these factors will be used to facilitate the development of protocol-based management of older people with hip fractures that is appropriate, context specific and acceptable by stakeholders in a low- and middle-income country setting, such as India.

**Electronic supplementary material:**

The online version of this article (doi:10.1186/s40814-016-0056-0) contains supplementary material, which is available to authorized users.

## Background

Fragility fractures in older adults, 60 years and over, caused by a low-impact injury, such as a minor fall, are often associated with osteoporosis and are one of the biggest healthcare challenges of the twenty-first century [1–3]. Among the fragility fractures, hip fractures are a major cause of morbidity and mortality [4]. Worldwide hip fractures are projected to increase from 1.7 million in 1990 to 6.3 million in 2050 [5, 6]. The projected increases are primarily the result of an ageing population and increased life expectancy, particularly in India and China [5]. In 2000, an estimated 9 million osteoporotic fractures occurred worldwide and the annual costs for treatment have been assessed to be around $20 billion in the USA and €30 billion in the European Union [7], with 72 % of this cost incurred for management of hip fractures. A report in 2004 estimated an annual incidence of 600,000 osteoporotic hip fractures in India [8], and this is expected to increase significantly as the percentage of people over 60 years rises from 5.6 % in 1961 to 12 % of 1.36 billion by 2026 [9]. Studies from India on bone health and hip fractures suggest that due to poor bone health and low socioeconomic status, osteoporotic hip fractures are likely to occur a decade earlier in Indian men and women compared to their western counterparts [10–12].

The length of stay in hospital is about 3 weeks in the UK—and half of elderly adults with hip fractures do not return to their usual place of residence [13]. Following a hip fracture, use of health services extends beyond the initial hospitalization for at least 1 year with much of healthcare costs attributable to subsequent long-term care [14–17]. In the UK, the 30-day and 1-year mortality following a hip fracture is 9 and 30 %, respectively [18]. According to studies from India, the crude incidence of hip fracture was 105 and 159 per 100,000 among men and women, respectively [19] and 1-year mortality was 42 % [4].

Protocol-based management of older adults with hip fractures was developed through large audits from Sweden [20, 21] and in the UK [13, 22–24]—countries with health systems providing universal healthcare with no cost at the point of delivery. These audits informed the development of best-practice guidelines and implementation as integrated care pathways (ICP) for management of older people with hip fractures in the UK and Sweden. The ICP comprise prompt admission to an orthopaedic ward, combined ortho-geriatrician care, surgery within 48 h of admission, geriatrician consultation within 72 h of admission, early post-operative mobilization, prevention of pressure ulcers, medication for osteoporosis, falls prevention and geriatrician led rehabilitation [20]. The introduction of ICP for management of older adults with hip fractures resulted in shorter length of hospital stay, earlier ambulation and fewer medical complications and pressure wounds, compared with a comparison group, and demonstrated 40 % reduction in cost of care [25, 26]. In addition, a systematic review of the effects of ICP-based management of hip fractures in high-income countries demonstrated reduction in hospital mortality and improvements in the organization of care [24].

There is little published information on the pathways of care and outcomes of individuals with fragility hip fractures in India. The two retrospective studies involving single hospital site suggest the mean age for hip fracture as 62 years with female predominance of 79 % and had no evidence on effectiveness of the services provided [4, 19]. If care is to be improved, firstly, we need to understand the current practice and management of older adults with hip fractures in India. Secondly, determine how and why these practices differ from best practice and outcomes globally and also determine potential barriers and facilitators relevant to reducing evidence-practice gaps. Thus, our proposed study will document current practice and determine appropriateness, acceptability and feasibility for adopting internationally accepted components of the ICP approach or protocol-based care [20] within the selected hospitals in an urban setting like Delhi. The study will also identify barriers to recruitment and retention of patients till the follow-up period. The aspects of the ICP reflecting pre-operative, operative and post-operative care for hip fracture management within the hospital will be explored using mixed methods in this study. The focus group discussion (FGDs) with healthcare providers (HCPs) in particular will seek information on current practices, multi-disciplinary collaboration, perceived barriers and facilitators to implementing the ICP.

## Methods

This will be a mixed-methods prospective cohort study. Qualitative methods will be used for exploring aspects of acceptability and appropriateness of the ICP, and mixed methods will be used to assess the feasibility of adopting the ICP in routine practice and barriers to implementation (Table 1).

**Table 1:** Aspects of acceptability, appropriateness and feasibility

Constructs	Methodology	Tools	Study dimension
Acceptability	Qualitative methods	KIIs and FGDs (HCP) FGDs (patient and their carers)	- Explore dimensions of the best-practice evidence for adoption/implementation, i.e. content, complexity and comfort from healthcare providers’ perspective - Patients’ perspectives on contextual (patient circumstances) and structural barriers (health setting)
Appropriateness	Qualitative methods	KIIs FGDs (HCP) FGDs (patient and their carers)	- To explore whether adopting evidence-based practice may be perceived as appropriate but not acceptable and vice versa - To capture barriers to implementation efforts - Patients’ perspectives on contextual (patient circumstances) and structural barriers (health setting)
Feasibility	Mixed methods (quantitative and qualitative)	Baseline survey questionnaire 30-day follow-ups KIIs and FGDs to identify barriers and facilitators	- Recruitment of study sites and patients - Response rate retention and follow-up rates - Determine specific outcome variables (primary and secondary) - Estimate sample size for the larger study - Identify barriers and facilitators for adopting ICP in routine practice - Barriers and facilitators for scale up

*KII* key informant interviews, *FGD* focus group discussion, *HCP* healthcare provider (consultants, registrars and nursing staff from the departments of orthopaedics, anaesthesia, geriatrics, medicine and physiotherapy)

**Fig. 1:**
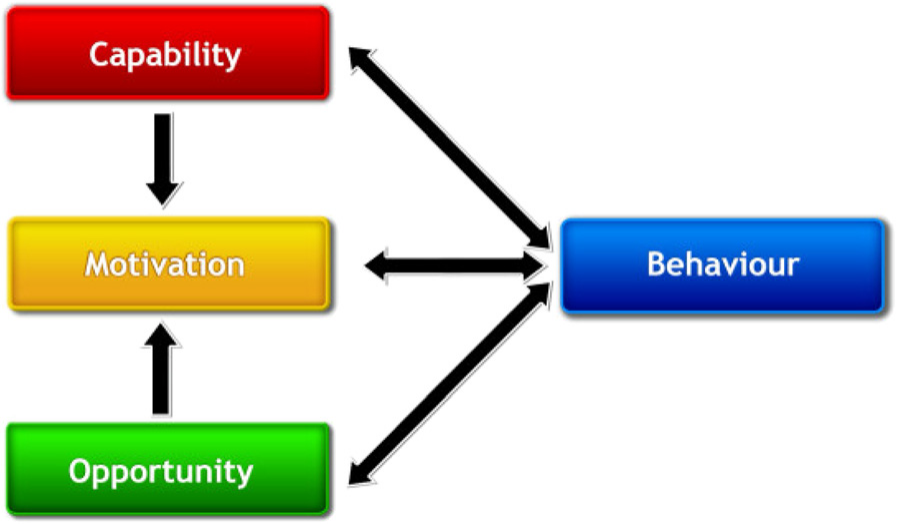
The COM-B system—a framework for understanding behaviour [27]

The study will also use the COM-B model (capability, opportunity, motivation and behaviour) [27] to explore stakeholders’ behaviour. The ‘COM-B’ model provides the framework to understand how capability, opportunity and motivation interact to generate behaviour that in turn influences these components and this knowledge will inform intervention strategies for successful implementation. Capability is defined in relation to an individual’s psychological and physical capacity to engage in the activity concerned. It includes having the necessary knowledge and skills. Motivation is defined as all those brain processes that energize and direct behaviour, not just goals and conscious decision-making. It includes habitual processes, emotional response as well as analytical decision-making. Opportunity is defined as all the factors that lie outside the individual that make the behaviour possible or prompt it (Fig. 1). The single-headed and double-headed arrows in Fig. 1 represent potential influences between components in the system. For example, opportunity can influence motivation as can capability; enacting behaviour can alter capability, motivation and opportunity.

### Study sites

The study will be undertaken at four hospital sites in Delhi, which is the second most populous city in India with a population of nearly 17 million. Prior to selection of these sites, stakeholder event was organized inviting representatives of major tertiary care hospitals in Delhi. The aims and objectives of the proposed study were discussed during this event and the sites that consented to participate were selected for the study. Thus, the study sites include three government centres and one private tertiary healthcare centre. The centres are as follows: the All India Institute of Medical Sciences (AIIMS), a national referral tertiary care centre with 1800 beds hospital under the Ministry of Health and Family Welfare; Jai Prakash Narayan Apex Trauma Centre (JPNATC), a comprehensive trauma centre and a national referral hospital; Guru Teg Bahadur Hospital, the associated teaching hospital of the University College of Medical Sciences (UCMS), a Constituent College of the University of Delhi, with 1000 beds, which receives patients from north Delhi and surrounding areas; and St Stephen’s Hospital, one of the largest private tertiary care hospitals in Delhi, with 600 beds, providing subsidized care for poor patients.

### Study participants

The participants for the qualitative component will be selected purposively and these will involve healthcare providers (HCPs), patients and their carers. HCPs will include clinical leads, residents (doctors in speciality training) and nursing staff from departments involved in the provision of care to older adults with hip fractures. These departments primarily will include orthopaedics, geriatrics, general medicine, anaesthesia, nursing and physiotherapy. Whereas for the quantitative component, all consecutive patients aged 50 years and above with an X-ray-confirmed hip fracture admitted and treated at the participating hospitals and those who provided consent will be recruited over a 2-month time period.

Key informant interviews (KIIs) and focus group discussions (FGDs) will be used as part of qualitative data collection.

#### Key informant interviews

The clinical leads of the departments involved in providing care to older people with hip fractures will be interviewed in order to understand existing care within their setting and their views on the barriers, facilitators and the feasibility of conducting a study aimed at implementing ICP for the management of hip fractures for older people. In total, 16 KIIs (four in each hospital) will be conducted with orthopaedic surgeons, geriatricians, anaesthetists, senior physiotherapists and nurses.

#### Focus group discussions

FGDs will be undertaken with HCPs, i.e. consultants, registrars and nursing staff from the departments of orthopaedics, anaesthesia, geriatrics, medicine and physiotherapy (Additional file 1). Two FGDs per site will be carried out with HCPs involved in pre-operative, operative and post-operative care. Additionally, patients operated in the past 3 months for a unilateral hip fracture and living in Delhi will be selected from the discharge register and invited to participate in a FGD along with their carer (Additional file 2). A total of eight FGDs will be conducted with patients and/or their carers or family members.

FGDs with HCPs will seek information on current practices, multi-disciplinary collaboration, perceived barriers and facilitators to implementing ICP using the COM-B model and issues related to recruitment and follow-up of patients. The FGDs with patients and carers will capture their perspective around the chronology of events leading to hospitalization, care needs, satisfaction and potential challenges and barriers to accessing treatment. Each group will have eight to ten participants and interviews will be conducted in English or Hindi, or both, as is appropriate.

Trained qualitative research staff will conduct these KIIs and FGDs and the team will be comprised of one moderator and one note-taker. The note-taker will take additional notes to supplement the analysis. All KIIs and FGDs will be tape-recorded, translated to English and transcribed for analysis.

#### Quantitative data collection

Patient data will be collected through a structured questionnaire administered to patients on admission to hospital, from medical records at discharge and through a brief telephone interview 30 days post fracture.

#### Inclusion criteria

Consecutive patients aged 50 years old or older, with an X-ray-confirmed hip fracture (ICD-10 code S72.0-S72.2) [28], admitted and treated at one of the participating hospitals will be recruited over a 2-month period.

#### Exclusion criteria

Patients with pathological fracture and terminal malignancies will be excluded from the study.

Key variables from the minimum dataset for the international hip fracture registry of the Fragility Fracture Network (FFN) have been utilized in the construction of the questionnaire. The FFN is a global network of activists with an aim to promote the optimal multidisciplinary management of the patients with a fragility fracture including secondary prevention. FFN maintains a literature registry of publications relating to the themes of FFN and serve as a one-stop global information hub for healthcare professionals, health system administrators, policy makers and payers from both public and private sectors [29]. These variables include the following:

#### On admission to hospital

Socio-demographic characteristics (age, sex, education, residence and occupation), information on the carer, information on known medical conditions or those diagnosed at the time of hospital admission and pre-fracture mobility.

#### At discharge

Data on pre-operative, peri-operative and post-operative care will be documented. This will include length of stay, in-hospital mortality and quality of life (QoL). Additional information such as mobility status, pressure sores, carer information and living arrangements will also be collected at discharge.

#### Thirty days post fracture

Data will be sought on mortality, complications, place of residence and mobility, and QoL will be measured using EQ-5D. The QoL will be measured by EQ-5D, a tool for measuring quality of life in terms of mobility, usual activities, anxiety/depression, self-care and pain/discomfort on a five-point scale [30].

### Data management and analysis

The electronic data will be kept password protected and stored on secure servers. The access will be allowed only to the key people involved in the research with an undertaking that the data will be solely used for the purpose of research.

The KIIs and FGDs will be analysed using Nvivo 9 software. Thematic analysis will be undertaken in order to identify major themes across the care pathway for the management of hip fractures in various hospitals (Table 1). Quantitative data will be analysed as per the outcome variables (Table 2). Findings from the quantitative survey of hospitalized patients will be corroborated with the statements recorded through FGDs and KIIs. Qualitative and quantitative data will be collected concurrently, such that weaknesses of one kind of data are ideally offset by strengths of the other kind. The qualitative and quantitative data will be analysed separately, and mixing will take place when the findings are interpreted using concurrent triangulation design. Important strengths of this approach are the ability to maximize the information provided by a single study [31].

**Table 2:** Key variables: quantitative data collection

Data collection	On admission	At discharge	30-day follow-up
Variables	- Care-seeking behaviour - Socio-demographic characteristics - Co-morbidities - Hospital service processes	- Pre-operative, operative and post-operative care - Complications	- Mobility^a^ - Functional status^a^ - Living arrangements - Re-admission/surgery
Best-practice indicators and outcomes	Time from injury to admission in A&E Time from A&E to orthopaedic ward	- Time from admission to assessment by physician/geriatrician - Time from admission to surgery - Discharged on bone protection medication - Received a falls assessment prior to discharge - Length of stay - Pressure ulcer - In-hospital mortality	- Quality of life^a^ - Caring needs - 30-day mortality

^a^EQ-5D, a tool for measuring quality of life in terms of mobility, usual activities, anxiety/depression, self-care and pain/discomfort on a five-point scale

### Ethical approval

Necessary permissions have been obtained from participating Institutional Ethics Committee (IEC) of AIIMS (vide letter; IEC/NP-40/13.03.2014, RP-32/2014), St. Stephens’ Hospital (vide letter; Minutes of Ethics Committee Meeting-30.04.2014, Proposal No.4), UCMS (vide letter 18.07.2014) and Health Ministry Screening Committee (HMSC) at Indian Council of Medical Research (vide letter no. 54/1/Indo-foreign/GER/2014-NCD-II, dated 10 October 2014).

## Discussion

The study proposes to explore the feasibility of implementing a protocol-based care pathway derived from best-practice guidelines to improve health outcomes of older adults with hip fractures in India. Implementation of practice that has demonstrable benefits in high-income countries, to a contextually different health system, introduces intrinsic challenges and understanding these contextual factors is of paramount importance. The study utilizes theoretical framework of implementation science to investigate significant factors which will inform contextually appropriate modifications of best practices for effective knowledge translation. The findings will enable appropriate strategies to design behaviour change interventions for successful implementation of care pathway for the management of older adults with hip fractures.

## Additional files

Additional file 1: Focus group discussion for healthcare providers. (DOCX 781 kb)
Additional file 2: Patient and carer focus group discussion. (DOCX 780 kb)
