# Supplementary material for: Protocol-based management of older adults with hip fractures in Delhi, India: a feasibility study
Source: Pilot Feasibility Stud. 2016 Mar 9;2:15. doi: 10.1186/s40814-016-0056-0 (PMC5154050; doi:10.1186/s40814-016-0056-0)
Supplement: Additional file 1: — Focus group discussion for healthcare providers. (DOCX 781 kb) [file 40814_2016_56_MOESM1_ESM.docx]

1. Please share your experiences about people with hip fracture in your daily practice?

*Probe: Profile of patients, your role as practitioner, available resources, expectations from hospitals& patient side.*

1. In your opinion how important is multi disciplinary care in hip fracture cases. Please list down the various discipline involved in such surgeries.
2. Please brief us about the usual protocol for care of hip fracture cases?

*Probe: availability of diagnostic(X-ray, MRI,CT scan etc.),timing of surgery including pre-operative assessment & care,type of analgesia,type of anaesthesia, planning of theatre team, surgical procedures, mobilisation and multidisciplinary management.*

1. Operating older people with hip fractures should be a priority? Please comment.
2. According to you , how many patients of hip fractures come to the hospital and how many gets operated?
3. In your opinion what kind of information is helpful to patients& their carer/family members? Generally, how this information is provided at the key stages of patient journey.

*Probe: Admission, early mobilization, pain control, rehabilitation, discharge & follow up prevention*

1. In your opinion are there any areas for improvement in management of hip fractures or care protocols?

*Probe: barriers & facilitators*

1. Any other suggestion?
