# Supplementary material for: Protocol-based management of older adults with hip fractures in Delhi, India: a feasibility study
Source: Pilot Feasibility Stud. 2016 Mar 9;2:15. doi: 10.1186/s40814-016-0056-0 (PMC5154050; doi:10.1186/s40814-016-0056-0)
Supplement: Additional file 2: — Patient and carer focus group discussion. (DOCX 780 kb) [file 40814_2016_56_MOESM2_ESM.docx]

Q:1 Please tell us about your experience at the time of admission to the hospital

Probe: 1) Condition at the time of first visit to the hospital; pain, mobility, discomfort, etc. 2) Admission Process

Q:2: Are you aware of any of the hospital staff who explained the condition and treatment options available for you? How useful that was in your opinion?

Probe: 1) Easy to understand; language 2) Elaborate or concise; T/t modalities, risks & outcomes 3) Opportunity to ask questions 4) Developed Confidence in decision making 5) Became more anxious & who helped you to overcome

Q:3: Tell us about your experience(or experience of the patient, in case carer is a respondent) during the stay at the hospital

Probe: 1) Post-operative care at ICU or high dependency units 2) Nursing care

Q:4: Can you tell us about some of the advice given as part of the post-operative care?

Probe: 1) Preventing pressure sores 2) Advantage of early mobilisation

Q:5: Please tell us about the total expenditure you have incurred during the stay at the hospital?

Q:6: Please provide us some suggestions on how the care within the hospital can be improved?
